# Quantitative Detection of Vertical Track Irregularities under Non-Stationary Conditions with Variable Vehicle Speed

**DOI:** 10.3390/s24123804

**Published:** 2024-06-12

**Authors:** Qiushi Wang, Hui Zhao, Dao Gong, Jinsong Zhou, Zhongmin Xiao

**Affiliations:** 1 Department of Military Traffic Injury Prevention and Control, Daping Hospital, Army Medical University, Chongqing 400042, China; qs_wang_1991@tmmu.edu.cn (Q.W.); zhaohui@tmmu.edu.cn (H.Z.); 2 Institute of Rail Transit, Tongji University, Shanghai 201804, China; gongdao@tongji.edu.cn; 3 School of Mechanical and Aerospace Engineering, Nanyang Technological University, Singapore 639798, Singapore

**Keywords:** railway vehicle, track irregularity, non-stationary, order tracking, quantitative detection

## Abstract

Track irregularities directly affect the quality and safety of railway vehicle operations. Quantitative detection and real-time monitoring of track irregularities are of great importance. However, due to the frequent variable vehicle speed, vehicle operation is a typical non-stationary process. The traditional signal analysis methods are unsuitable for non-stationary processes, making the quantitative detection of the wavelength and amplitude of track irregularities difficult. To solve the above problems, this paper proposes a quantitative detection method of track irregularities under non-stationary conditions with variable vehicle speed by order tracking analysis for the first time. Firstly, a simplified wheel–rail dynamic model is established to derive the quantitative relationship between the axle-box vertical vibration and the track vertical irregularities. Secondly, the Simpson double integration method is proposed to calculate the axle-box vertical displacement based on the axle-box vertical acceleration, and the process error is optimized. Thirdly, based on the order tracking analysis theory, the angular domain resampling is performed on the axle-box vertical displacement time-domain signal in combination with the wheel rotation speed signals, and the quantitative detection of the track irregularities is achieved. Finally, the proposed method is validated based on simulation and field test analysis cases. We provide theoretical support and method reference for the quantitative detection method of track irregularities.

## 1. Introduction

Track irregularity is one of the main reasons for vibration in railway vehicle operation [1]. When these vibrations are unacceptable, several problems will follow, such as riding comfort reduction, running stability decline, wheel–rail noise pollution, fatigue strength failure of components, etc. [2,3,4,5]. Therefore, track irregularity should be controlled and managed as much as possible to create a better vehicle operating environment.

The current hot research topic of track irregularity can be summarized as the following aspects: ① Track infrastructure evolution—due to the coupling effect of temperature, humidity, load, and other factors, the railway infrastructure undergoes the evolution of physical and chemical properties. The track infrastructure presents overall or local cumulative deformation, which affects the track irregularity state [6,7,8]. ② Track-vehicle interaction—taking the track and vehicle as a whole system, the long-term interaction between the track and the vehicle will affect the track geometry. The deterioration of track irregularity will make the vehicle system produce a more significant response, accelerating the deterioration of track and vehicle components [9,10,11]. ③ Track management and maintenance—however, before the causing mechanisms of the problem mentioned above are thoroughly clarified, and some basic solutions are proposed, scientifically managing all kinds of railway (high-speed, heavy-haul, urban subways, etc.) operating lines and maintaining a safe, stable, and reliable railway vehicle service environment is a crucial problem [12,13].

The track management and maintenance prerequisite is to obtain actual track irregularity state data. The selection of measurement methods greatly affects the authenticity of the results. ① Static measurement—is directly measured by hand or using some small devices to obtain the track irregularity state data for a short distance. Since the force acting on the track by the measuring device is relatively small, the elastic deformation of the rail, bed, roadbed, etc., is negligible. The measured track irregularity data is geometric irregularity at this time, which is called static irregularity. ② Dynamic measurement—however, when the vehicles are running on the track, the enormous dynamic load can cause comprehensive elastic deformation of the rail, bed, and roadbed, called dynamic irregularity. Strictly speaking, compared with static irregularity, dynamic irregularity is more reflective of the actual vehicle working environment because it includes both the geometric irregularity before loading and track elastic deformation after loading [14,15,16]. Obviously, dynamic irregularity can not be obtained by direct measurement. Currently, two main indirect methods for detecting dynamic vertical track irregularity are as follows: the chord measurement method and the inertial reference method.

① The chord measurement method—takes the fixed chord length as the measurement reference and equal interval sampling is used to obtain the chord measurement value of each sampling point. Its mathematical calculation model is as follows [17]:

(1) $$y\left(k\right)=h\left(k\right)-\frac{\left[h\left(k-p\right)+h\left(k+p\right)\right]}{2}$$

where *k* is the mileage position of the sample point, *y*(*k*) is the chord measurement value at the mileage position *k*, *p* is the half chord length, and *h*(*k*), *h*(*k* − *p*), *h*(*k* + *p*) are the vertical irregularity amplitudes of the track at the mileage positions *k*, *k* − *p*, *k + p*.

Performing the discrete Fourier transform to Equation (1), the transfer function *H*(*ω*) can be expressed as:

(2) $$H\left(\omega \right)=\frac{y\left(\omega \right)}{h\left(\omega \right)}=\left[1-\frac{e^{-j\omega p}+e^{j\omega p}}{2}\right]=1-\cos\left(\omega p\right)$$

where *ω* is the spatial angular frequency (*ω* = 2π/*λ*), and *λ* is the track irregularity wavelength).

As shown in Equation (2), *H*(*ω*) is the transfer function related to the spatial angular frequency, independent of the vehicle running speed and time. Therefore, there is no non-stationary problem. However, the value of the transfer function *H*(*ω*) is varied with *ω* in the range [0, 2] and cos(*ωp*) is in the range [−1, 1] [18]. Especially when the value of the transfer function *H*(*ω*) is close to 0, it is easy to be disturbed by the noise signal, and the track irregularity state near its corresponding wavelength is difficult to detect, leading to a lack of stability in the measurement accuracy. In addition, the chord measurement method is only applied to a specific rail inspection vehicle. With the increasing growth of rail transportation tasks, the free time of rail inspection vehicles for line inspection is insufficient, and it is impossible to achieve real-time monitoring. Hence, the chord measurement method’s shortcomings in the economy and efficiency are gradually exposed [19,20,21].

② The axle-box on-board measurement method—due to the small elastic deformation caused by the interaction between the wheel and rail, the wheel and rail can be considered rigid bodies, and there is no suspension system between the wheelset and axle-box. All the track irregularity can be reflected in the axle-box vibration signal [22,23,24,25]. Therefore, evaluating the track irregularity state by collecting axle-box vibration information online has become a hot research topic in recent years. Kawasaki et al. obtained the acceleration vibration features of an axle-box by establishing a rail–vehicle system simulation model and then judged the track irregularity state through the features identification [26]; M. Bocciolon et al. developed a real-time track condition monitoring technique by calculating the mean square value of axle-box vibration acceleration to analyze the track irregularity state [27]; Yang et al. applied wavelet transform theory to eliminate the axle-box vibration acceleration trend term, performing the double integration to the acceleration signal and evaluating the track irregularity state based on the obtained axle-box displacement signal [28]; Ning et al. combined the improved empirical mode decomposition (EMD) with Cohen’s kernel, and applied it to the analysis of the axle-box non-stationary vibration signal to achieve online monitoring of the track irregularity fault location [29]; Zhang et al. designed a refined system transfer function identification test method by combining the iterative analysis of simulation and test to reproduce the track irregularity based on the axle-box vibration acceleration [30]; Sun et al. used the inertial reference method and digital filtering method to analyze the axle-box vibration acceleration of high-speed trains to achieve online monitoring of track irregularity [17]. However, the above studies were limited in solving the fundamental problem of non-stationary vibration signals, and have not been able to achieve quantitative detection of the essential parameters of the track irregularity.

**Figure 1:**
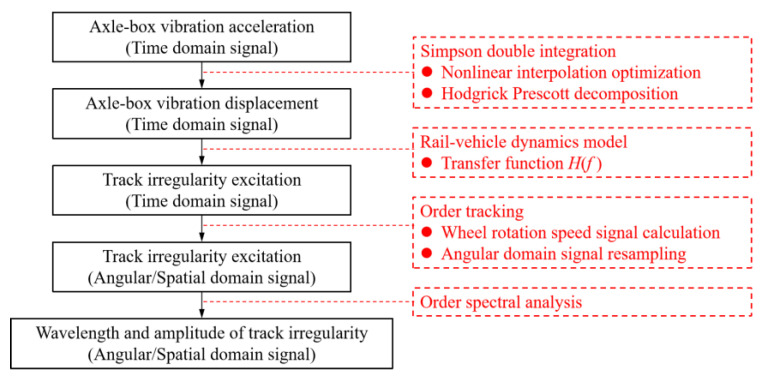
The overall technical route.

This paper proposes a complete set of quantitative detection methods for track irregularities under variable vehicle speed conditions. In Section 1, a brief overview of the current research about track irregularities is given. In Section 2, a simplified wheel–rail dynamics model is established, and the quantitative relationship between axle-box vertical vibration frequency, amplitude, and track irregularity is demonstrated by theoretical derivation. In Section 3, the axle-box vertical vibration displacement calculation method based on Simpson time-domain double integration is proposed, and the improved Hodrick–Prescott decomposition method is used to optimize the process error. Section 4 proposes the quantitative detection method of track vertical irregularity under vehicle variable speed conditions based on the order tracking analysis theory. In Section 5 and Section 6, the correctness of the above quantitative detection method is verified based on simulation and field test cases, respectively. The conclusion is given in Section 7. The overall technical route is shown in Figure 1.

## 2. The Quantitative Relationship between the Axle-Box Vertical Vibration and Vertical Track Irregularity

**Figure 2:**
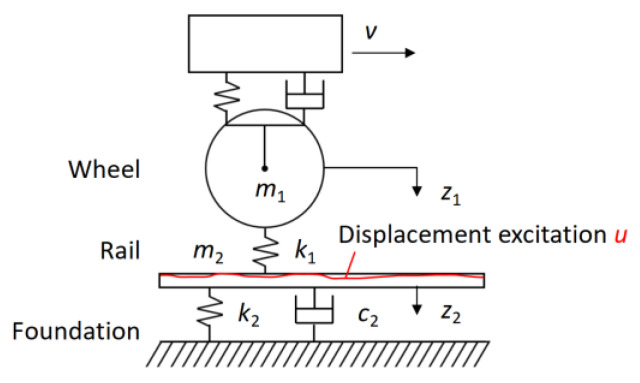
Vertical wheel–track dynamic model.

To derive the quantitative relationship between the axle-box’s vertical vibration and vertical track irregularity, a simplified vertical wheel–rail dynamics model is established, as shown in Figure 2. Due to the minimal elastic deformation caused by the interaction between the wheel and rail, the dynamic model can be considered a rigid body [31,32]. Because there is no suspension system between the wheelset and axle-box, the track irregularity characteristics can be reflected in the vibration signal of the axle-box.

In Figure 2, *v* is the vehicle running speed, *m*_1_ is the unsprung mass, *m*_2_ is the mass of the steel rail, *k*_1_ is the elastic contact stiffness between the wheel and rail, *k*_2_ is the vertical stiffness of the rail, *c*_2_ is the vertical damping of the rail, *z*_1_ is the vertical displacement of the wheelset/axle-box, *z*_2_ is the vertical displacement of the steel rail, and *u* is the vertical displacement excitation. The directions to the right and down are positive here.

The motion differential equation of the above dynamic model is expressed as:

(3) $$\left\{\begin{array}{l} m_{1}{\ddot{z}}_{1}\left(t\right)+k_{1}\left[z_{1}\left(t\right)-u\left(t\right)-z_{2}\left(t\right)\right]=0\\ m_{2}{\ddot{z}}_{2}\left(t\right)+c_{2}{\dot{z}}_{2}\left(t\right)+k_{2}z_{2}\left(t\right)-k_{1}\left[z_{1}\left(t\right)-u\left(t\right)-z_{2}\left(t\right)\right]=0 \end{array}\right.$$

When the initial displacement and velocity are both 0, the Laplace transform of Equation (3) can be written as:

(4) $$\left\{\begin{array}{l} \left(\frac{m_{1}s^{2}}{k_{1}}+1\right)z_{1}\left(s\right)-z_{2}\left(s\right)=u\left(s\right)\\ z_{1}\left(s\right)-\left(\frac{m_{2}s^{2}}{k_{1}}+\frac{c_{2}s}{k_{1}}+\frac{k_{2}}{k_{1}}+1\right)z_{2}\left(s\right)=u\left(s\right) \end{array}\right.$$

**Figure 3:**
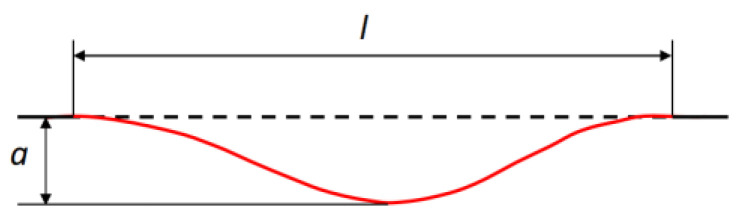
Single harmonic irregularity excitation.

The irregularity excitation can be seen as a linear superposition of multiple harmonic waves with different wavelengths, amplitudes, and phases. The irregularity excitation *u*(*t*) of a single harmonic, as shown in Figure 3, can be expressed as a cosine function:

(5) $$u\left(t\right)=\frac{1}{2}a\left[1-\cos\left(\omega t\right)\right]$$

where *a* is the wave depth of the irregularity and *ω* is the time angular frequency.

By the Laplace transformation, Equation (5) can be rewritten as:

(6) $$u\left(s\right)=\frac{a\omega^{2}}{2s\left(s^{2}+\omega^{2}\right)}$$

Then, to find the transfer function of the responses *z*_1_ and *z*_2_ corresponding to the irregularity excitation *u*, assume the following relationship:

(7) $$\left\{\begin{array}{l} z_{1}\left(s\right)=H_{z_{1}}\left(s\right)u\left(s\right)\\ z_{2}\left(s\right)=H_{z_{2}}\left(s\right)u\left(s\right) \end{array}\right.$$

where *z*_1_(*s*) and *z*_2_(*s*) are the Laplace transforms corresponding to *z*_1_(*t*) and *z*_2_(*t*), and *H_z_*_1_(*s*) and *H_z_*_2_(*s*) are the transfer functions corresponding to the irregularity excitation *u*(*s*) in response to *z*_1_(*s*) and *z*_2_(*s*).

By combining Equations (4) and (7), the transfer function *H_z_*_1_(*s*) can be solved:

(8) $$H_{z_{1}}\left(s\right)=\frac{m_{2}s^{2}+c_{2}s+k_{2}}{\frac{m_{1}m_{2}s^{4}}{k_{2}}+\frac{m_{1}c_{2}s^{3}}{k_{1}}+\left[\left(\frac{k_{2}}{k_{1}}+1\right)m_{1}+m_{2}\right]s^{2}+c_{2}s+k_{2}}$$

Then, the quantitative relationship between the axle-box vertical vibration acceleration and the vertical irregularity excitation can be expressed as:

(9) $${\ddot{z}}_{1}\left(s\right)=s^{2}z_{1}\left(s\right)=s^{2}H_{z_{1}}\left(s\right)\;u\left(s\right)$$

Then, the vertical vibration acceleration and displacement of the axle-box can be expressed as:

(10) $${\ddot{z}}_{1}\left(s\right)=\frac{1}{2}a\omega^{2}\left|H_{z_{1}}\left(s\right)\right|\frac{s}{s^{2}+\omega^{2}}$$

(11) $$z_{1}\left(s\right)=\frac{{\ddot{z}}_{1}\left(s\right)}{s^{2}}=\frac{1}{2}a\omega^{2}\left|H_{z_{1}}\left(s\right)\right|\frac{1}{s\left(s^{2}+\omega^{2}\right)}$$

Solving the inverse Laplace transformation of Equations (10) and (11), the axle-box vertical vibration acceleration and displacement can be obtained:

(12) $${\ddot{z}}_{1}\left(t\right)=\frac{1}{2}a\omega^{2}\left|H_{z_{1}}\left(j\omega \right)\right|\cos\left(\omega t\right)$$

(13) $$z_{1}\left(t\right)=\frac{1}{2}a\left|H_{z_{1}}\left(j\omega \right)\right|\left[1-\cos\left(\omega t\right)\right]$$

Comparing the oscillation characteristic terms in Equations (12) and (13), the amplitude of the vertical acceleration of the axle-box is *ω*^2^ times the vertical displacement with a phase difference π. According to the vertical displacement *z*_1_(*t*) of the axle-box and the frequency response function *H_z_*_1_(j*ω*), the vertical track irregularity excitation can be calculated, as shown in Equations (7) and (8). With the vehicle running speed *v* known, the vertical vibration frequency of the axle-box can be used to estimate the wavelength of the track irregularity. The vertical vibration amplitude *D* of the axle-box acceleration or displacement can be used to estimate the wave depth of the track irregularity.

## 3. Time-Domain Track Vertical Irregularity Displacement Excitation

Equation (12) can be written as:

(14) $${\ddot{z}}_{1}\left(t\right)=D\cos\left(\omega t+\psi \right)+Q_{0}$$

where *D* is the amplitude of axle-box vertical vibration acceleration, which is a constant factor related to *ω*_1_, *ω*_2_, *c*_2_, *k*_1_, *k*_2_, and *ω*. *ψ* is the phase and *Q*_0_ is the constant term.

Then, the vertical vibration velocity and displacement of the axle-box can be obtained by the first and second times integrals of Equation (14), expressed as:

(15) $${\dot{z}}_{1}\left(t\right)=\frac{D}{\omega }\sin\left(\omega t+\psi \right)+Q_{0}t+Q_{1}$$

(16) $$z_{1}\left(t\right)=-\frac{D}{\omega^{2}}\cos\left(\omega t+\psi \right)+Q_{0}t^{2}+Q_{1}t+Q_{2}$$

Comparing the oscillation characteristic term in Equations (15) and (16), the amplitude of the vertical acceleration of the axle-box is *ω*^2^ times the vertical displacement with a phase difference π. This characteristic is entirely consistent with the conclusion in Section 2, which indicates that it is theoretically possible to use the double integration method to calculate the vertical oscillation displacement of the axle-box. Then, combining the frequency response function *H_z_*_1_(j*ω*), the track irregularity excitation can be calculated.

### 3.1. Improved Simpson Time-Domain Integration Method

There are two methods for calculating displacement by using integrated acceleration: the frequency-domain method and the time-domain method. However, the railway vehicle speed in operation frequently changes. The vibration signal is the typical non-stationary signal. The traditional discrete Fourier transform will lead to frequency ambiguity in the frequency domain, leading to serious estimation errors. Therefore, the frequency domain integration method is unsuitable for analyzing and processing this situation. However, the time-domain integration can avoid frequency distortion, frequency ambiguity, and other problems caused by discrete Fourier transform [33,34]. The Simpson integral formula can be expressed as:

(17) $$\int_{a}^{b}f\left(x\right)\;dx\approx \frac{1}{6}\left(b-a\right)\left[f\left(a\right)+4f\left(\frac{a+b}{2}\right)+f\left(b\right)\right]$$

where *a* and *b* represent the lower and upper limits of the integration interval, respectively.

Substituting the discrete acceleration time series *a*(*n*), (*n* = 1, 2, …, *N*_0_) into Equation (18) can obtain the velocity time series *v*(*n*), (*n* = 1, 2, 3, …, *N*_0_ − 1).

(18) $$\left\{\begin{array}{l} v\left(1\right)=\frac{a\left(1\right)+a\left(2\right)}{2}\Delta t\\ v\left(2\right)=v\left(1\right)+\frac{a\left(1\right)+4a\left(2\right)+a\left(3\right)}{6}\Delta t\\ \ldots \\ v\left(n\right)=v\left(n-1\right)+\frac{a\left(n-1\right)+4a\left(n\right)+a\left(n+1\right)}{6}\Delta t \end{array}\right.$$

Then, substitute the discrete acceleration time series *v*(*n*), (*n* = 1, 2, …, *N*_0_ − 1) into Equation (19) to obtain the displacement time series *s*(*n*), (*n* = 1, 2, 3, …, *N*_0_ − 2).

(19) $$\left\{\begin{array}{l} s\left(1\right)=\frac{v\left(1\right)+v\left(2\right)}{2}\Delta t\\ s\left(2\right)=s\left(1\right)+\frac{v\left(1\right)+4v\left(2\right)+v\left(3\right)}{6}\Delta t\;\\ \ldots \\ s\left(n\right)=s\left(n-1\right)+\frac{v\left(n-1\right)+4v\left(n\right)+v\left(n+1\right)}{6}\Delta t \end{array}\right.$$

where Δ*t* is the time interval.

Furthermore, the integral remainder error of Simpson formula can be expressed as:

(20) $$R\left[f\right]=-\frac{b-a}{180}{\left(\frac{b-a}{2}\right)}^{4}f^{\left(4\right)}\left(\eta \right)\;,\;\eta \in \left(a,b\right)$$

Equation (20) shows that the residual error of the Simpson formula is related to (b − a). The absolute value of the error decreases as the integration range [a, b] between adjacent points decreases. Therefore, we propose to add a nonlinear interpolation to the initial signal before integration to reduce the time interval of the signal and, thus, improve the integration accuracy. This idea is validated in Section 3.3.

### 3.2. Trend Term Decomposition and Elimination

In addition to the integration errors, the errors caused by the trend term should be given more attention. Since the initial value of the acceleration signal is affected by uncertain factors during the acquisition process, the signal is often accompanied by phenomena such as zero drift and noise interference. This error information accumulates and amplifies during integration, affecting the calculation results. Removing these trend terms is a critical issue in time-domain integration operations.

The main trend term elimination methods are polynomial fitting, CEEMDAN empirical mode decomposition, and Hodrick–Prescott trend decomposition. The research on polynomial fitting and CEEMDAN empirical mode decomposition is quite mature, and their theories and methods can be seen in references [35,36,37], which do not need to be elaborated further. This paper will focus on the theory and application of the Hodrick–Prescott trend decomposition method and compare it with the above two methods to verify its correctness.

Assuming there is a trend term *g*(*n*) and a residual term *c*(*n*) in the signal *a*(*n*):

(21) $$a\left(n\right)=g\left(n\right)+c\left(n\right)$$

where *a*(*n*) represents the initial signal, *g*(*n*) represents the trend term, and *c*(*n*) represents the residual term, which contains the fluctuation and noise terms.

Then, establish a loss function, which is expressed as:

(22) $$M=\sum_{n=1}^{N_{a}}{\left[a\left(n\right)-g\left(n\right)\right]}^{2}+\lambda \sum_{n=2}^{N_{a}}{\left[g\left(n+1\right)-2g\left(n\right)+g\left(n-1\right)\right]}^{2}$$

where *λ* is the smoothing factor.

As shown in Equation (22), the first term on the right is essentially the principle of least squares, characterizing the closeness between the initial signal *a*(*n*) and the trend term *g*(*n*). The second term on the right can be viewed as a second-order difference equation for *g*(*n*), i.e., Δ*g*(*n*) − Δ*g*(*n* − 1) = [*g*(*n +* 1) − *g*(*n*)] − [*g*(*n*) − *g*(*n* − 1)], characterizing the smoothness of the desired trend term *g*(*n*). When the loss function *M* takes the minimum value, it means that the resulting trend term *g*(*n*) is both closest to the trend of the initial signal *a*(*n*) and sufficiently smooth. 

Calculate the first order derivatives of Equation (22) to *g*(1); *g*(2); …, respectively, and set them equal to 0:

(23) $$\left\{\begin{array}{l} \frac{\partial M}{g\left(1\right)}=-2\left[a\left(1\right)-g\left(1\right)\right]+2\lambda \left[g\left(1\right)-2g\left(2\right)+g\left(3\right)\right]=0\\ \frac{\partial M}{g\left(2\right)}=-2\left[a\left(2\right)-g\left(2\right)\right]+2\lambda \left[-2g\left(1\right)+5g\left(2\right)-4g\left(3\right)+g\left(4\right)\right]=0\\ \;\ldots \\ \frac{\partial M}{g\left(i\right)}=-2\left[a\left(i\right)-g\left(i\right)\right]+2\lambda \left[g\left(i-2\right)-4g\left(i-1\right)+6g\left(i\right)-4g\left(i+1\right)+g\left(i+2\right)\right]=0\\ \;\ldots \\ \frac{\partial M}{g\left(n\right)}=-2\left[a\left(n\right)-g\left(n\right)\right]+2\lambda \left[g\left(n-2\right)-2g\left(n-1\right)+g\left(n\right)\right]=0 \end{array}\right.$$

It can be expressed as matrix form:

(24) A−G=C=λFG

where ***A*** is the initial signal vector, ***G*** is the trend term vector, ***C*** is the residual term vector, and ***F*** is the coefficient matrix, expressed as Equation (25).

(25) F=1−21      −25−41     1−46−41     1−46−41     ⋱⋱⋱⋱⋱     1−46−41     1−46−41     1−45−2      1−21

Therefore, the formula for the trend term vector ***G*** can be expressed as:

(26) $$G=A{\left(\lambda F+I\right)}^{-1}$$

where ***I*** is the unit vector.

As shown in Equation (26), the trend term *g*(*n*) can be separated from the initial signal *a*(*n*) as long as the smoothing factor *λ* takes an appropriate value. When the loss function *M* is a number very close to 0, the optimal smoothing factor *λ* can be expressed as:

(27) $$\lambda =-\frac{\sum_{n=1}^{N_{a}}{\left[c\left(n\right)\right]}^{2}}{\sum_{n=2}^{N_{a}}{\left[g\left(n+1\right)-2g\left(n\right)+g\left(n-1\right)\right]}^{2}}\approx -\frac{\mathrm{var}\left[c\left(n\right)\right]}{\mathrm{var}\left[\Delta^{2}g\left(n\right)\right]}=-\frac{\sigma_{c}^{2}}{\sigma_{\Delta^{2}g}^{2}}$$

where *σ_c_*^2^ is the variance of the residual term *c*(*n*) and $\sigma_{\Delta^{2}g}^{2}$ is the variance of the second-order difference of the trend term *g*(*n*).

Since the trend term *g*(*n*) and the residual term *c*(*n*) are unknown before the calculation, the smoothing factor *λ* is also unknown. Here, we propose an optimal smoothing factor *λ* search method, as the following technical route:

(a) consider the smoothing factor as a coefficient to be determined and set the initial value as *λ_j_* (*j* = 0);
(b) substitute *λ_j_* into Equation (26) to separate the trend term *g_j_*(*n*) and the residual term *c_j_*(*n*);
(c) calculate the variance $\sigma_{\Delta^{2}g}^{2}$ of the second-order difference of the trend term *g_j_*(*n*) and the variance *σ_cj_* ^2^ of the residual term cj(n), respectively;
(d) calculate the equivalent error *ε_j_*, *ε_j_ =* |−*λ_j_σ*_Δ*gj*_^2^/*σ_cj_*^2^| − 1;
(e) reset *λ_j_ = λ_j_* + *j*Δ*λ* (*j* = 1, 2, …) and repeat the steps (a) to (d);
(f) find *λ_j_* corresponding to the smallest equivalent error *ε_j_*, which is the optimal smoothing factor *λ_opt_*.

In addition, it is worth noting that the optimal smoothing factor *λ_opt_* determined by the above technical route must be based on certain assumptions, namely that the trend term *g*(*n*) and the residual term *c*(*n*) are subordinate to independent normal distributions. Meanwhile, the second-order differential equation for *g*(*n*) in Equation (22) shows that the determination of the smoothing factor is closely related to the sampling time interval. Therefore, it has been proposed that the smoothing factor should be corrected according to the sampling frequency (or sampling interval).

**Figure 4:**
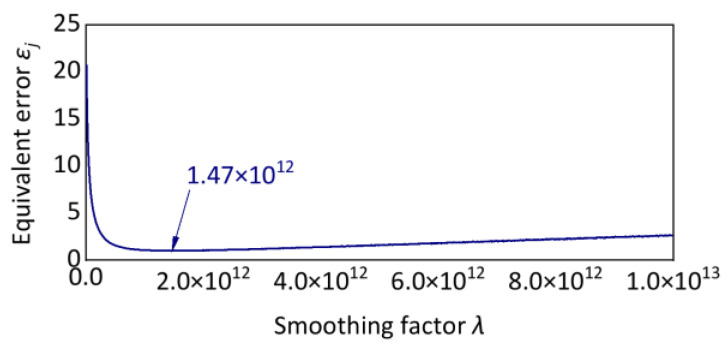
Searching for the optimal smoothing factor.

However, the correction method is controversial, and the formula has no exact derivation and proof [38,39,40]. In this paper, a series of simulated signals were cyclically tested. The fitted trend curves were analyzed for different values of the smoothing factor. It was proposed that the fitted trend curve for a vibration signal with a sampling frequency of 5120 Hz and *λ* = 1.47 × 10^12^ has the smallest error with the simulated trend curve, as shown in Figure 4.

### 3.3. Validation and Analysis

To verify the correctness of the above time-domain integration method and trend decomposition method, assume a simulation vibration acceleration signal *a*(*t*) and use the above methods to calculate the vibration displacement. The simulation signal *a*(*t*) is superimposed by five signals. *a*_1_(*t*), *a*_2_(*t*), and *a*_3_(*t*) represent the vibration acceleration generated by short, medium, and long wave periodic track irregularities features, respectively:

(28) $$a\left(t\right)=a_{1}\left(t\right)+a_{2}\left(t\right)+a_{3}\left(t\right)+a_{4}\left(t\right)+a_{5}\left(t\right)$$

where *a*_1_(*t*) = 30·cos(2π·50*t* + 60) and *a*_2_(*t*) = 10·cos(2π·6*t* + 120); *a*_3_(*t*) = 2·cos(2π·2*t* + 201).

Then, to express the non-stationary characteristics, assume the signal *a*(*t*) contains an initial trend term *a*_4_(*t*):

(29) $$a_{4}\left(t\right)=\left\{\begin{array}{cc} 50t+35 & t\leq 0.5\\ 20t^{2}+30t+40 & 0.5<t\leq 1\\ 20t^{3}+10t^{2}-40t+100 & 1<t\leq 2 \end{array}\right.$$

Finally, add a Gaussian white noise signal *a*_5_(*t*) into the signal *a*(*t*); its mean is 0, and its variance is 100. Perform the analogue-to-digital conversion to the analogue signal and obtain the discrete signal *a*(*n*). The sampling time is 2 s and the sampling frequency is 500 Hz.

**Figure 5:**
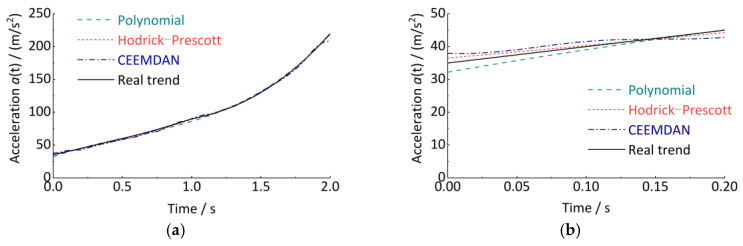

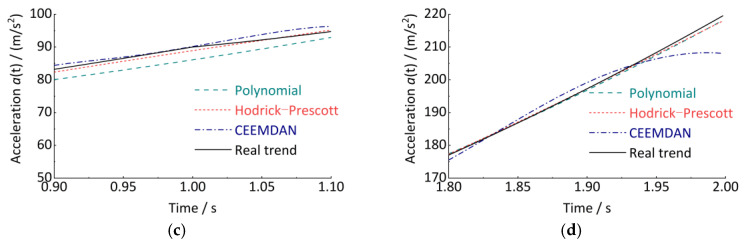
Fitting of the trend term. (**a**) Time interval 0 s~2 s (overall); (**b**) Time interval 0 s~0.2 s (partial); (**c**) Time interval 0.9 s~1.1 s (partial); (**d**) Time interval 1.8 s~2.0 s (partial).

The three trend term decomposition methods are used for the analysis, as shown in Figure 5. All three methods as a whole can fit the trend term well. However, the polynomial fit decomposition method has a poor fit superiority at the breakpoints of the segmentation function (e.g., 0 s, 1.0 s). The CEEMDAN empirical modal decomposition method has serious trend errors at the end, caused by the inherent defect of the empirical modal decomposition class of methods (i.e., the endpoint error effect). However, the Hodrick–Prescott decomposition method has the most stable overall performance. This is because the Hodrick–Prescott decomposition method contains both the least squares fitting idea, which makes the fitted trend term closest to the initial signal, and the fluctuation term constraint, which makes the fitted trend term smooth enough.

**Figure 6:**
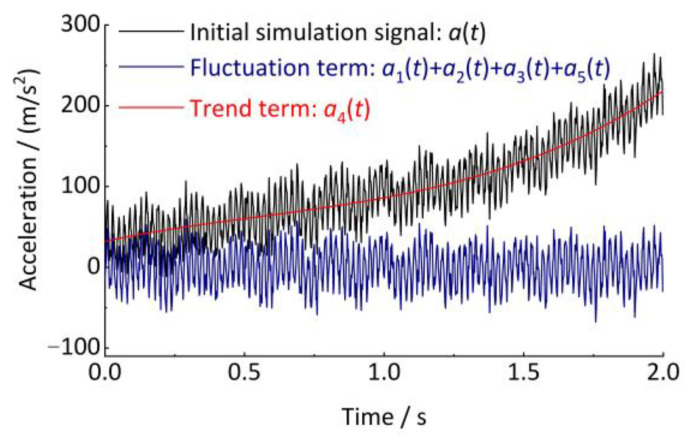
Decomposition of trend item.

As shown in Figure 6, both the single-segment and segmented trend curves show good adaptability. Therefore, the Hodrick–Prescott decomposition method is also used in the subsequent integration calculation for trend elimination of the curves before and after integration.

**Figure 7:**
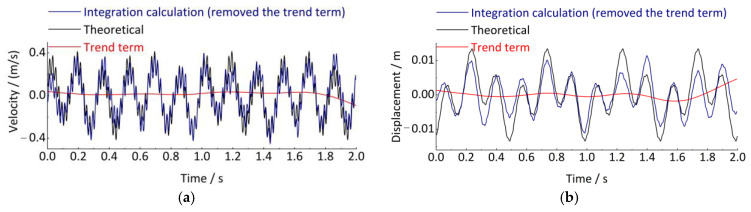
Simpson time-domain integral calculation. (**a**) Speed signal; (**b**) Displacement signal.

Then, as shown in Equations (17)–(19), the Simpson double integration method can be used to calculate the axle-box vertical displacement based on the acceleration signal. It is necessary to perform three trend term eliminations on the signal before the first integration, before the second integration, and after the second integration. Taking the simulation acceleration signal *a*(*t*) as an example, the results obtained by the first and second direct Simpson integrations are shown in Figure 7 for the velocity signal and the displacement signal, respectively. The time-domain integration process produces the trend terms, and the velocity and displacement signals after removing the trend term have a reduced error with the theoretical signal.

**Figure 8:**
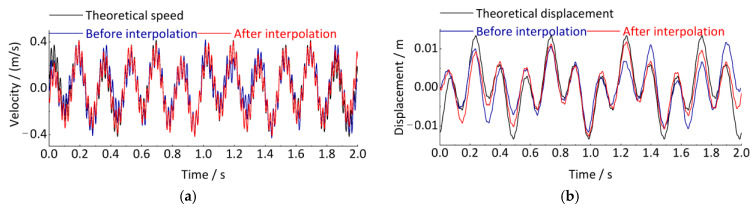
Compare the calculation results of interpolation and non-interpolation before integration. (**a**) Speed signal; (**b**) Displacement signal.

Meanwhile, since the error is related to the size of the integration region [a, b] between adjacent points, theoretically, the error is smaller when [a, b] is smaller. We propose to reduce the sampling interval of the signal by adding a spline interpolation step before the integration and then improve the accuracy of the time-domain integration. As shown in Figure 8, the Simpson integration error can indeed be effectively reduced by reducing the sampling time interval of the signal, improving the accuracy of the time-domain integration. 

To quantitatively evaluate the accuracy of the interpolated and uninterpolated methods before time-domain integration, the degree of agreement between the theoretical displacement signal and the displacement signal obtained by integration is reflected by calculating the correlation index R. The closer the value of R is to 1, the higher the accuracy of the integration calculation [41]. The correlation index R is 0.7343 for the displacements calculated directly without interpolation before integration, while the correlation index R is 0.9210 for the displacements calculated after interpolation before integration, and the correlation is significantly improved.

In summary, it is theoretically possible to calculate the axle-box vertical displacement signal by double integration of the axle-box vibration acceleration. Considering that the measured signal is non-stationary, the time-domain integration method should be used, and the effects of integration error and trend term error should be focused on. Finally, the time-domain track vertical irregularity displacement excitation can be calculated according to the axle-box vertical displacement and the frequency response function *H*(*ω*).

## 4. Quantitative Detection of Vertical Track Irregularities Based on the Order Tracking Analysis Theory

Railway vehicles always operate at variable speeds, causing the axle-box vertical vibration acceleration to become the typical non-stationary signal. Hence, the time-domain track vertical irregularity displacement excitation calculated according to the method in Section 3 is the non-stationary signal. Traditional signal processing methods cannot detect the wavelength and amplitude of track irregularities from non-stationary signals. But, if these time-domain signals can be converted into the spatial domain signal, the problem of non-stationary signals will be completely solved. Considering the wheel as the rotating mechanical component, we propose the order tracking analysis theory for the non-stationary signal processing for the first time. This section will focus on the detailed derivation and verification of applying this theory in the quantitative detection of vertical track irregularities.

### 4.1. Introduction and Derivation of Order Tracking Analysis Theory

Order tracking analysis is an effective method for extracting fault features under variable speed conditions. The key is to measure and analyze the wheel rotational speed or angular velocity, then resample the time-domain signal non-uniformly by using an equal-angle sample method. Therefore, the non-stationary signal in the time-domain is transformed into a stationary signal in the angular domain (spatial domain). Finally, the resampled angular-domain signal is analyzed by order spectrum to detect the fault features of the components quantitatively.

The order is a signal characterization parameter related to wheel rotational speed, expressed as:

(30) $$o=\frac{60\times f}{R}$$

where *o* is the order (dimensionless), *f* is the vibration frequency, *R* is the rotational speed of the wheel (revolutions per minute, rpm), and *R* = 60·*ω*/2π; *ω* is the angular velocity of the wheel.

As shown in Equation (30), the order tracking analysis is essentially a kind of frequency analysis. However, the frequency information is not fixed; it changes with the rotational speed change. The larger the order *o*, the higher the corresponding vibration frequency *f*, which reflects the vibration characteristics of short wave track irregularities. When the order *o* is smaller or even the fractional (a number less than 1), the corresponding vibration frequency *f* is lower, which reflects the vibration characteristics of long-wave vertical track irregularities.

**Figure 9:**
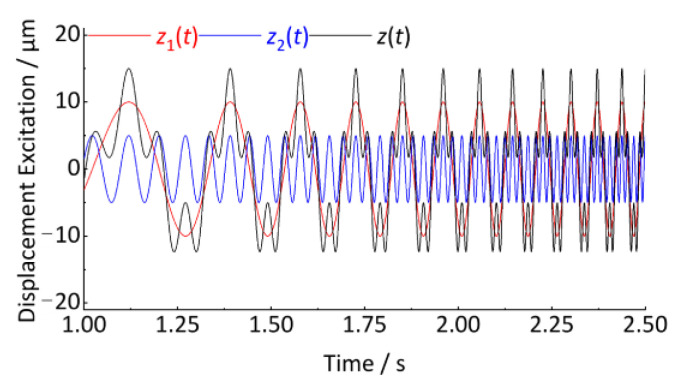
Simulated non-smooth signal (partial).

A simple case is used to illustrate how to convert the time-domain track vertical irregularity displacement excitation into a spatial signal in the angular domain, and finally, the order spectrum analysis is performed. Let a track vertical irregularity displacement excitation be *z*(*t*), as shown in Equation (31) and Figure 9. The sampling frequency is 1024 Hz and the total sampling length is 10 s.

(31) $$z\left(t\right)=z_{1}\left(t\right)+z_{2}\left(t\right)=10\cdot \cos\left(2\pi \left(t^{3}-0.5t^{2}+0.2t\right)\right)+5\cdot \cos\left(2\pi \left(4t^{3}-2t^{2}+0.8t\right)\right)$$

The signal *z*(*t*) contains two vibration features *z*_1_(*t*) and *z*_2_(*t*), having the characteristic of increasing vibration frequency with time, which is typical of a non-stationary signal. These vibration features in Figure 9 are similar to the axle-box vertical irregularity displacement excitation generated when rail vehicles accelerate on tracks. *z*_1_(*t*) and *z*_2_(*t*) correspond to the wavelength of *l*_1_ and *l*_2_ of the two kinds of periodic vertical track irregularity.

**Figure 10:**
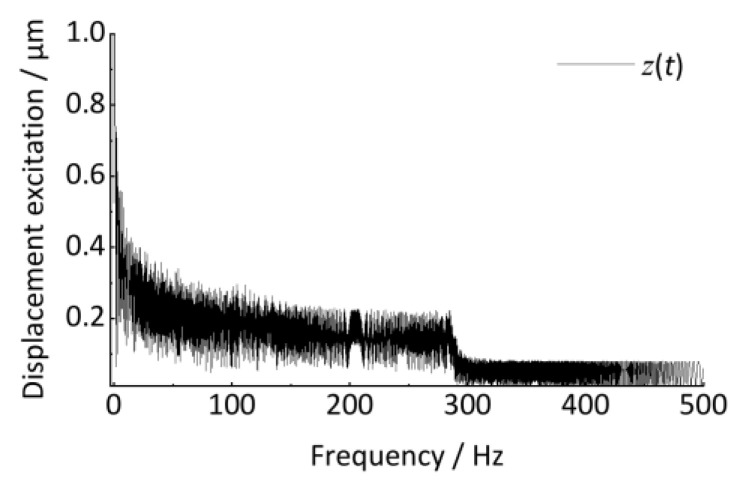
Traditional frequency spectrum analysis.

**Figure 11:**
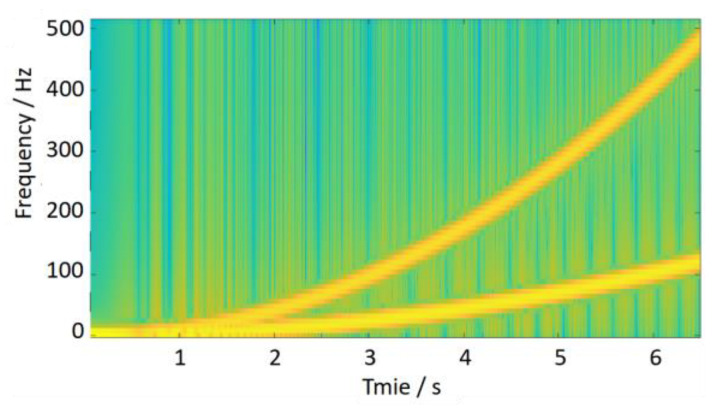
Traditional time–frequency analysis.

If the discrete Fourier transform is directly applied to estimate the irregularity characteristics (wavelength, amplitude) from the signal, *z*(*t*) will cause serious errors, even incorrect results. As shown in Figure 10, it exhibits severe frequency ambiguity with characteristic frequencies ranging from 0 to 500 Hz. The wavelength and amplitude of irregularities are completely unrecognizable. As shown in Figure 11, When the time–frequency analysis method is used, the change in signal frequency with time is analyzed by a short-time Fourier transform. It exhibits the trend of frequency variation in the time-frequency plot. However, the magnitude of the track irregularity is reflected by the depth of the color and does not quantitatively express the degree of track irregularity. In addition, the frequency ridges are also very blurred (thick), which will cause significant errors in estimating the frequency (wavelength). Therefore, traditional frequency domain analysis methods are unsuitable for the above non-stationary signals.

However, the above problems will be very clear when the order tracking analysis is used. Basically, the vertical displacement excitation frequency is related to the vehicle speed and the wavelength, i.e., *f* = *v/l*. Since the vertical track irregularity *l* is fixed and unchanged, the ultimate influence on the vibration frequency *f* is the operating vehicle speed *v*. Due to the mutual conversion relationship between the vehicle running speed *v* and the wheel rotation speed *R*, the complete derivation process is as follows:

(32) $$f=\frac{v}{l}=\frac{\omega \cdot r}{l}=\frac{\frac{2\pi R}{60}r}{l}=\frac{\frac{2\pi r}{60}R}{l}=\frac{\frac{C}{60}R}{l}=\frac{R\frac{C}{l}}{60}=\frac{Ro}{60}$$

where *f* is the vertical displacement excitation frequency (axle-box vertical vibration frequency), *v* is the vehicle running speed (wheel longitudinal speed), *ω* is the wheel angular velocity, *r* is the wheel radius, *l* is the vertical track irregularity wavelength, *C* is the wheel circumference, *o* is the order (the ratio of the wheel circumference *C* to the track vertical irregularity wavelength *l*, *C*/*l*), and *R* is the wheel rotation speed.

Therefore, according to the simulated signal in Equation (31), its wheel rotational speed can be calculated:

(33) $$R_{1}\left(t\right)=\frac{60\cdot f_{1}\left(t\right)}{o_{1}}=\frac{60}{o_{1}}\left(t^{2}-0.5t+0.2\right)$$

(34) $$R_{2}\left(t\right)=\frac{60\cdot f_{2}\left(t\right)}{o_{2}}=\frac{4\cdot 60}{o_{2}}\left(t^{2}-0.5t+0.2\right)$$

**Figure 12:**
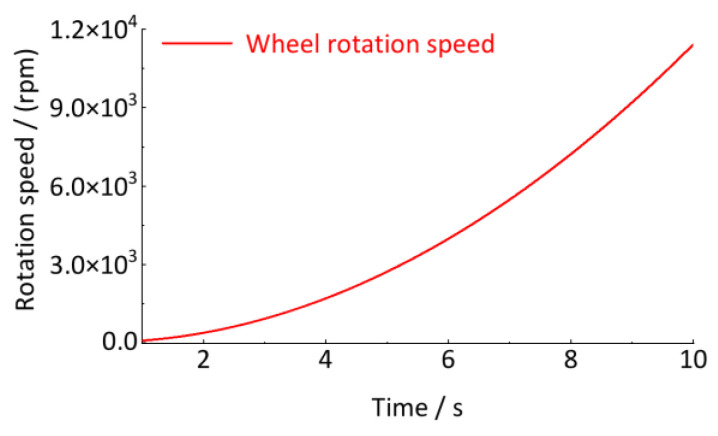
Wheel rotation speed curve.

Since the vibration response characteristics of *z*_1_(*t*) and *z*_2_(*t*) are generated by the same wheel in operation, the wheel rotational speed must be the same, i.e., *R*(*t*) = *R*_1_(*t*) = *R*_2_(*t*). Different vibration characteristics actually correspond to different track irregularity characteristics. As shown in Equations (33) and (34), *o*_1_ = *o*_2_/4, which is expressed as the wavelength *l*_1_ is four times *l*_2_, i.e., *l*_1_ = 4*l*_2_. In this case, assuming the wheel circumference *C* = 2*l*_2_, there are orders *o*_1_ = 1/2, *o*_2_ = 2. Therefore, the signals *z*_1_(*t*) and *z*_2_(*t*) essentially represent the physical meaning of the vibration response generated by a wheel traveling on a track with two periods of irregular characteristics at a speed of *R*(t). The wavelength of these two periods of irregularity is one-half of the wheel circumference, i.e., *l*_2_ = *C*/2; one is two times the wheel circumference, i.e., *l*_1_ = 2*C*. The wheel rotational speed is shown in Figure 12.

### 4.2. Resample the Time-Domain Signal Non-Uniformly in the Equal-Angle Sample Method

Once the wheel rotation speed time-domain signal is obtained, the original time signal can be resampled non-uniformly in the equal-angle sample method. Before that, we summarize some parameter relationships for order tracking analysis. When the length of the sampled data is an integer multiple *P* of the arc length of the wheel rotation, and the number of sample points for each revolution of the wheel is *M*, the maximum analysis order *O_max_* in the order domain is *M*/2, and the order analysis resolution Δ*O* is 1/*P*. In other words, *M* is the sampling rate and *M* × *P* is the sampling length. The larger the value of *M*, the wider the order domain of the analysis. The larger the value of *P*, the higher the accuracy of the order domain of the analysis.

Since the order *o*_1_ = 1/2, *o*_2_ = 2 in this case, according to Nyquist’s sampling theorem, the sampling points number for each wheel revolution should be *M* = 2·max(*o*_1_, *o*_2_) = 4. Meanwhile, like the traditional frequency domain analysis, the order domain analysis also exists the phenomenon of signal aliasing, caused by the sampling interval Δ*θ* being too large or the sampling rate *M* being too small, which will cause serious estimation errors. There are two ways to solve the signal aliasing problem. ① An anti-aliasing filter—using the low-pass filter to clear the frequency components larger than half of the sampling frequency can avoid signal aliasing problems. However the actual filter hard has the ideal filter characteristics, and it is easy to introduce artificial errors. ② Higher sampling rate— increasing the sampling rate or decreasing the sampling interval can solve the signal aliasing problems. In this case, since *M* = (2.56~4)·max(*o*_1_, *o*_2_), let *M* = 8 and Δ*θ* = 2π/8 = π/4. Finally, considering the order analysis resolution Δ*O =* 1/2 can guarantee that the order spectrum does not suffer from spectrum value leakage. Therefore, *P* = 2^n^. In this case, let *P* = 2, which means the signal must be collected for two revolutions of the wheel.

**Figure 13:**
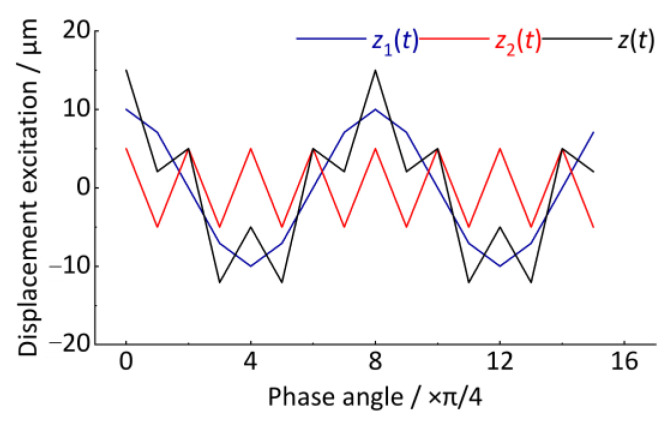
Angular domain signal *z*(*t*) (*M* = 8, *P* = 2).

After resampling the *z*(*t*) signal, the angular domain signal is shown in Figure 13. The horizontal coordinate is the wheel rotation phase angle, Δ*θ* = π/4. The vertical coordinate is the vertical irregularity displacement excitation whose amplitude remains unchanged at 10 μm and 5 μm. Compared with the *z*(*t*) signal in Figure 9, it shows good stationarity after converting the time-domain signal to the angular domain signal.

### 4.3. Order Spectrum Analysis and Quantitative Detection

**Figure 14:**
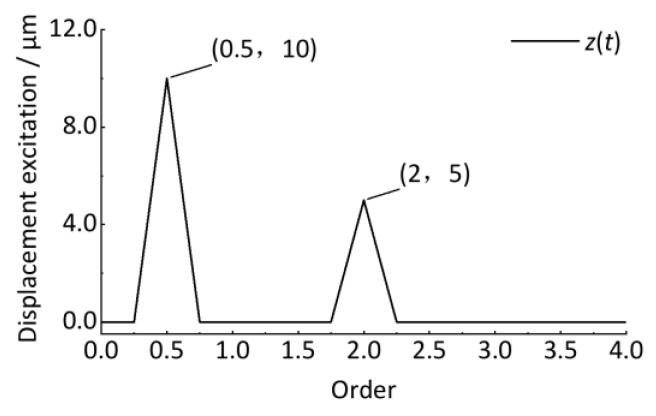
The order spectrum (*M* = 8, *P* = 2).

Then, the discrete Fourier transform is performed to obtain the order spectrum, as shown in Figure 14. The horizontal coordinate is the order that reflects the ratio of the wheel circumference C to the track vertical irregularity wavelength, as shown in Equation (32). The vertical coordinate is the amplitude. Compared with Figure 10 and Figure 11, the amplitude of the corresponding vibration can be clearly identified with 0 error, proving the advantage of order tracking analysis to the non-stationary signal whose vibration frequency is related to the wheel rotational speed, as shown in Figure 13 and Figure 14. In practical engineering, since the exact wavelength of the track irregularity is not known at the beginning, it is difficult to ensure that the collected signal corresponds to an integer multiple of the period irregularity, causing the signals to be non-periodic. If the discrete Fourier transform is directly performed on these non-periodic signals, it will cause energy leakage in the order spectrum. For this case, like the traditional frequency domain analysis, the window function (Hanning) can be used to the resampled angular domain signal, and then correcting the spectrum value of the discrete Fourier transformed spectrum by multiplying 2.0, the desired result can be obtained.

Based on the order information, it can be converted into a spatial domain signal of track vertical irregularity:

(35) $$u\left(x\right)=10\cdot \cos\left(2\pi \frac{x}{l_{1}}\right)+5\cdot \cos\left(2\pi \frac{x}{l_{2}}\right)=10\cdot \cos\left(2\pi \frac{xo_{1}}{C}\right)+5\cdot \cos\left(2\pi \frac{xo_{2}}{C}\right)$$

where *u* is the vertical track irregularity, *x* is the longitudinal distance of the track, *l*_1_ and *l*_2_ are the track irregularity wavelength, *C* is the wheel circumference, and *o*_1_ and *o*_2_ are the orders.

**Figure 15:**
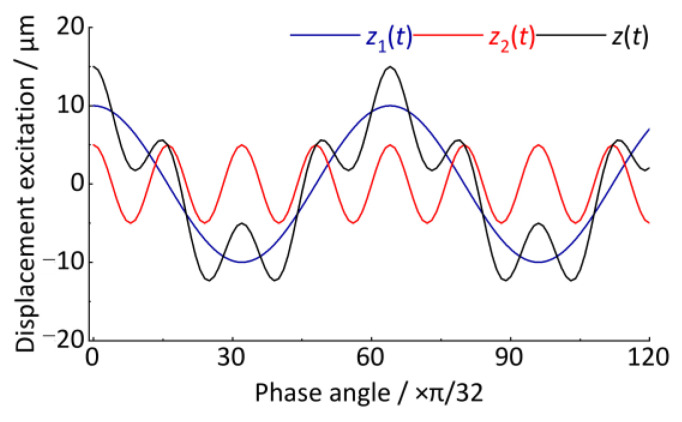
Angular domain signal *z*(*t*) *(M* = 64, *P* = 2).

**Figure 16:**
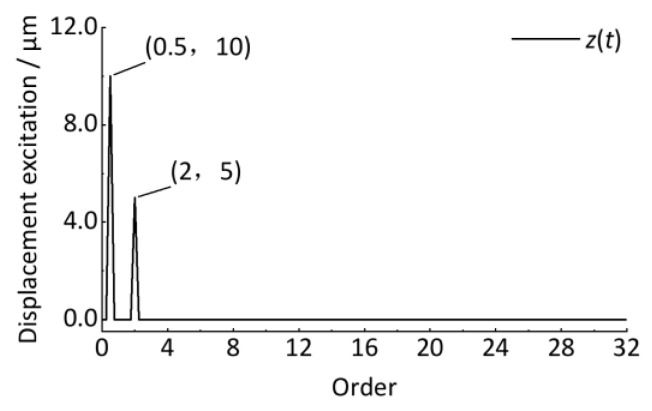
The order spectrum (*M* = 64, *P* = 2).

**Figure 17:**
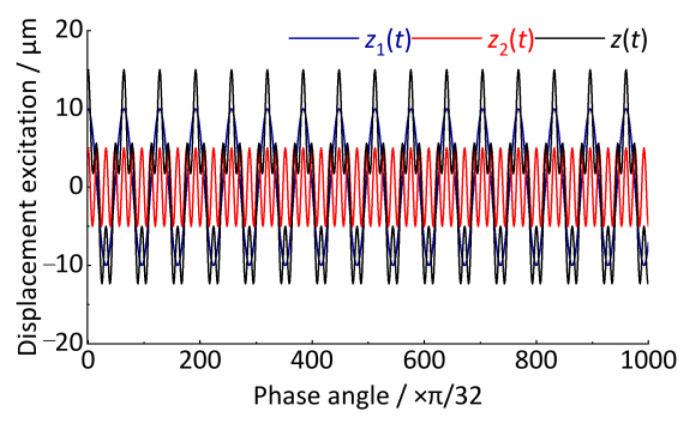
Angular domain signal *z*(*t*) (*M* = 64, *P* = 8).

**Figure 18:**
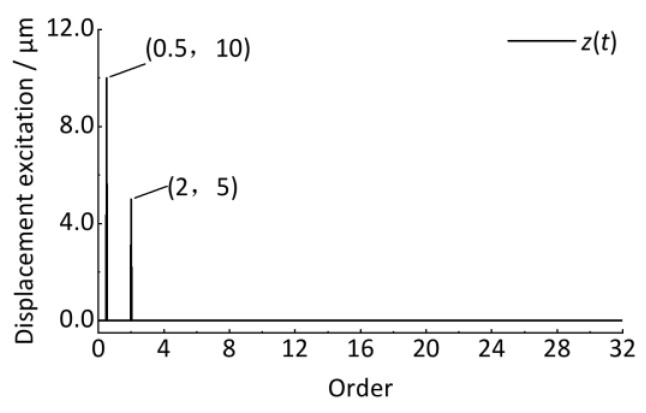
The order spectrum (*M* = 64, *P* = 8).

The curves in Figure 13 could be smoother, mainly because the *M* and *P* determined in the above cases are smaller enough, which satisfies the analysis accuracy requirement. Compared with Figure 13 and Figure 14, as shown in Figure 15, Figure 16, Figure 17 and Figure 18, with increasing *M*, the sampling point number of each wheel revolution will increase, and the sampling interval Δ*θ* will decrease, resulting in a smoother curve. With the increase of *P*, the number of sampled rotations will increase, resulting in a longer signal. Therefore, to detect short-wave track irregularity, we should try to make *M* larger. Increasing the *P* value will achieve a better order analysis resolution, which will help to distinguish the two types of track irregularity with similar wavelengths in the order spectrum.

## 5. Simulation Case Analysis and Verification

To verify the accuracy and reliability of the above methods, this section conducts simulation analysis and verification by establishing a vehicle system dynamics model.

### 5.1. Track–Vehicle Dynamic Model

**Figure 19:**
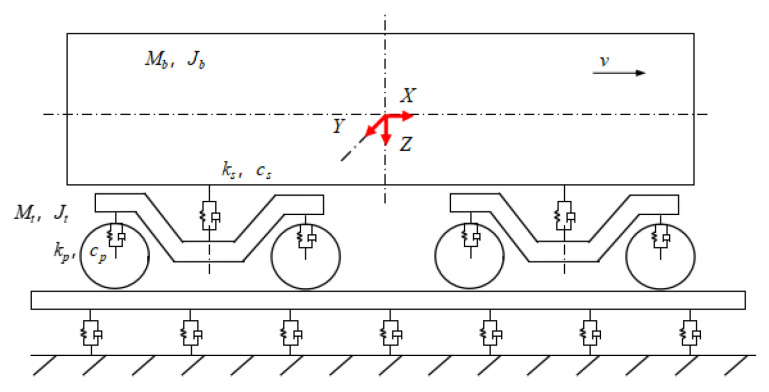
Track–vehicle vertical dynamic model. where *M_b_* is the body mass, *M_t_* is the bogie frame mass, *J_b_* is the body nodding inertia, *J_t_* is the bogie frame nodding inertia, *k_s_* is the secondary spring vertical rigidity, *k_p_* is the primary spring vertical rigidity, *c_s_* is the secondary spring vertical damping, and *c_p_* is the primary spring vertical damping.

Due to the weak coupling between the vertical and lateral dynamics of rail vehicles, the modeling can be carried out independently to reduce the complexity of the problem. The vehicle vertical dynamics model is shown in Figure 19.

**Figure 20:**
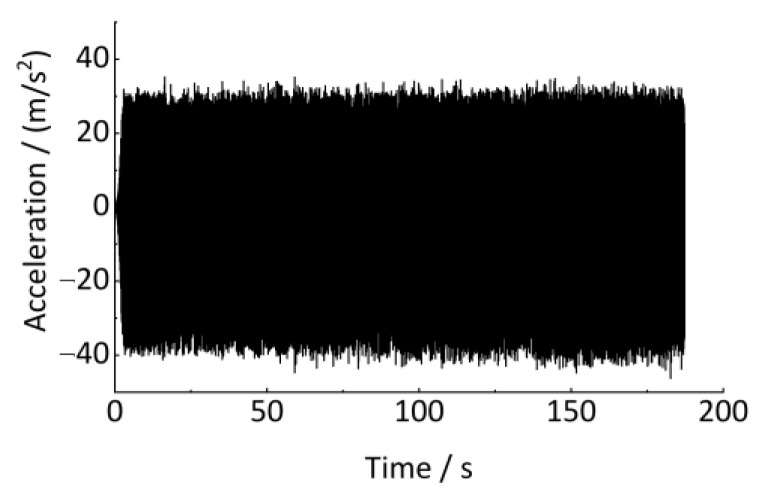
Axle-box vertical vibration acceleration.

**Figure 21:**
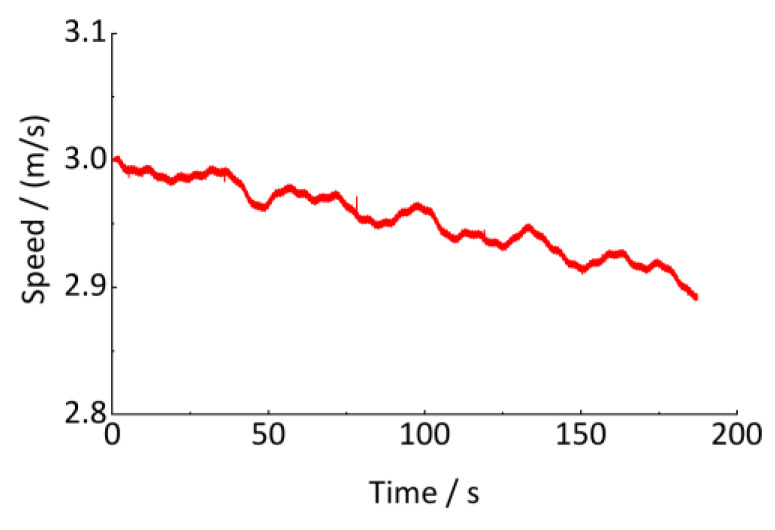
Vehicle running speed.

As shown in Table 1, a series of track vertical irregularities are designed and are input as the excitation in the above model. The wheel circumference is 2639 mm. The vehicle runs on a 1000 m straight track with an initial speed of 10.8 km/h. The axle-box vertical vibration acceleration and the vehicle running speed are output to estimate the track irregularities. The sampling frequency is 1000 Hz, and the sampling time is 190 s, as shown in Figure 20 and Figure 21.

### 5.2. Signal Analysis and Pre-Processing

**Figure 22:**
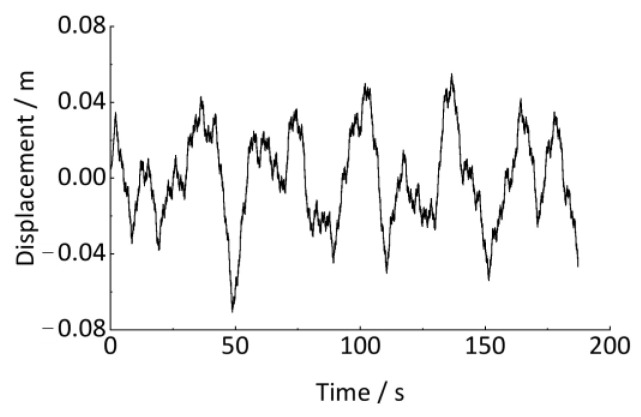
Axle-box vertical vibration displacement.

The axle-box vertical vibration displacement time-domain signal can be obtained by time-domain double integration and trend term elimination, according to the method in Section 3, as shown in Figure 22. Since the low vehicle speed in this validation scheme, the transfer relationship between the axle-box vertical displacement and the track vertical irregularity displacement excitation is close to 1. Therefore, the axle-box vertical displacement basically follows the track vertical irregularity at this time.

**Figure 23:**
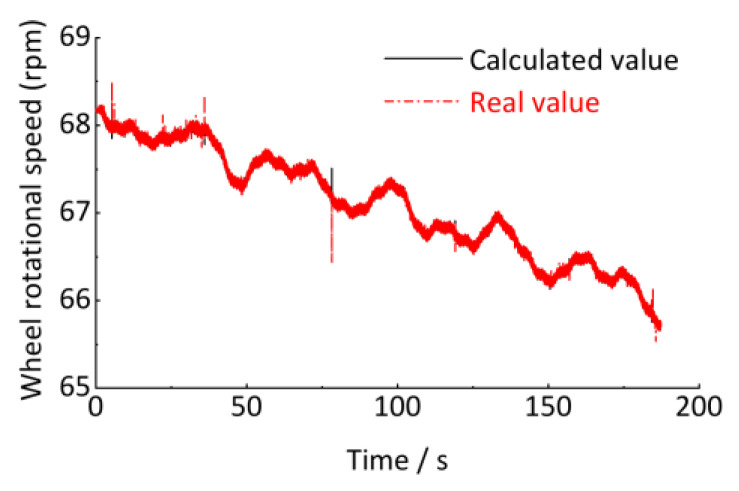
Vehicle running speed.

However, as shown in Figure 21, the vehicle speed frequently changes during operation. The axle-box vertical displacement signal in Figure 22 is non-stationary. As shown in Figure 10 and Figure 11, traditional signal processing methods cannot quantitatively detect the wavelength and amplitude of track irregularities from non-stationary signals. However, the time signal in Figure 22 can be resampled non-uniformly in the equal-angle sample method and converted into the angular (spatial) domain signal by using the proposed method in Section 4. The quantitative detection of track irregularities can be achieved. According to the analysis method in Section 4, it is known that the estimation of the wheel rotational speed *R* is the key to converting the time-domain signal into the angular domain signal. The relationship between the wheel rotational speed *R* and the vehicle running speed *v* is shown in Equation (32), and the wheel rotational speed is calculated, as shown in Figure 23.

Based on the improved Simpson integration method and trend item decomposition method in Section 3, the wheel rotation phase angle time-domain signal can be calculated as shown in Equation (36). Then, determine the time *t_n_* (*n* = 0, 1, 2, …) according to the wheel rotation phase angle (*θ_n_* = *n* · Δ*θ*, *n* = 0, 1, 2, …). Finally, we sequentially extract the corresponding values from the axle-box vertical displacement time-domain signal (Figure 22) based on the time *t_n_* (*n* = 0, 1, 2, …) to obtain the resampled signal.

(36) $$\left\{\begin{array}{l} \theta \left(1\right)=\frac{2\pi }{60}\frac{R\left(1\right)+R\left(2\right)}{2}\Delta t\\ \theta \left(2\right)=\theta \left(1\right)+\frac{2\pi }{60}\frac{R\left(1\right)+4R\left(2\right)+R\left(3\right)}{6}\Delta t\\ \ldots \\ \theta \left(n\right)=\theta \left(n-1\right)+\frac{2\pi }{60}\frac{R\left(n-1\right)+4R\left(n\right)+R\left(n+1\right)}{6}\Delta t \end{array}\right.$$

where *θ_n_* is the wheel rotation phase angle time series, and *R*(*n*) is the wheel rotational speed time series.

In the above case study, since the wavelength characteristics of track irregularity, as shown in Table 1, combined with the wheel circumference and vehicle running speed, it is necessary for the order spectrum analysis to accurately express the characteristics of track irregularity when Δ*O* ≤ 0.01 and *M* ≥ 4 × 52.779 (i.e., *P* ≥ 50, *M* ≥ 211.11). Therefore, let *P* = 100 and *M* = 240 here to ensure sufficient accuracy. That is, collect the vibration signal corresponding to 100 revolutions of the wheel with resampled at equal angular intervals for 240 points per revolution (i.e., Δ*θ* = 2π/*M* = π/120). However, all the above signals are discrete (non-continuous) and cannot be expressed by specific functions. When resampling by equal angles, the time *t_n_* (*n* = 0, 1, 2, …) may not exist or between two discrete time points, causing the sampling failure. Therefore, this paper proposes a nonlinear interpolation method to obtain an accurate value between two discrete points as follows.

**Figure 24:**
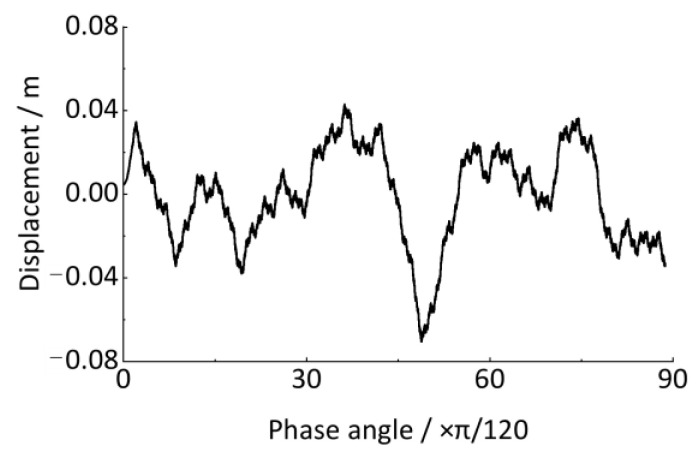
Resampled angular domain signal of axle-box vertical vibration displacement (*M* = 240, *P* = 100).

Assuming a cubic piecewise polynomial function *S*(*t*) that passes through *t*_1_, *t*_2_, *t*_3_, and *t*_4_, to calculate the *S*(*t_n_*) (*t*_2_ < *t_n_* < *t*_3_), the analytical expression of the function *S*(*t*) needs to be solved. Therefore, 12 conditional formulas must be needed to determine the 12 polynomial coefficients in total: ① the *S*(*t*_1_) = *B*_1_, *S*(*t*_2_) = *B*_2_, *S*(*t*_3_) = *B*_3_, and *S*(*t*_4_) = *B*_4_ are already known by simulated output signal; ② based on the fact that *S*(*t*) has a continuous second derivative and satisfies continuity conditions at *t*_2_ and *t*_3_, i.e., $S(t_{j-0})=S(t_{j+0}),\dot{S}(t_{j-0})=\dot{S}(t_{j+0}),\ddot{S}(t_{j-0})=\ddot{S}(t_{j+0}),j=2,3$; ③ there are two endpoint boundary conditions are already known by simulated output signal, i.e., $\dot{S}(t_{1})=C_{1},\dot{S}(t_{4})=C_{4}$. We solve the cubic piecewise polynomial *S*(*t*) based on the above 12 conditional formulas, and substitute *t* = *t_n_* into it to obtain *S*(*t_n_*). The resampled angular domain signal of the axle-box vertical vibration displacement is shown in Figure 24, which is the spatial domain signal and does not have non-stationary characteristics.

### 5.3. Order Spectrum Analysis

**Figure 25:**
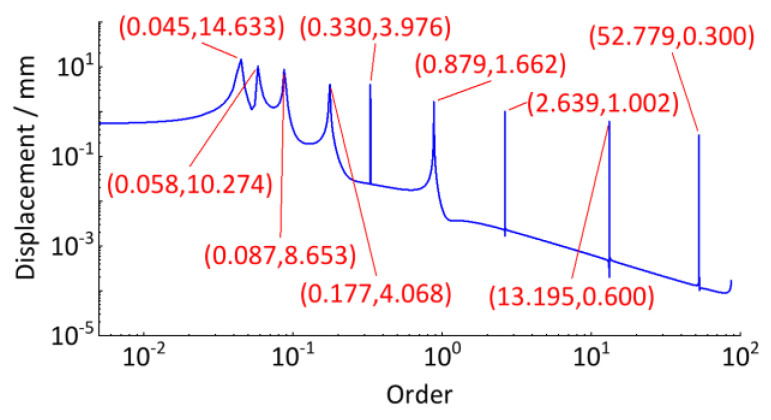
Order analysis spectrum of track irregularity.

We perform the discrete Fourier transform to the angle-domain signal in Figure 24 to obtain the order spectrum. As shown in Figure 25, the horizontal axle represents the order, which is expressed as the ratio of the wheel circumference to the track irregularity wavelength. The vertical axle represents the amplitude of the track irregularity. Compared with the real amplitude in Table 1, the wavelength estimation error of track irregularity is less than 2.31%, and the amplitude estimation error of the track irregularity is less than 20%, as shown in Table 2.

As shown in Table 2, the accuracy of the order analysis in estimating the short and medium wave irregularity is significantly higher than the long wave irregularity. The main reasons are as follows: ① since the long wave irregularity generally corresponds to low frequency signals, which are easily susceptible to interference from trend terms. Because the trend term can not be completely eliminated, even a small residual error will affect the estimation accuracy; ② due to the wide variety of track irregularity wavelengths, it is impossible to ensure the intercepted signal is an integral multiple of the period of corresponding wavelength irregularity. There must be obvious truncation errors when analyzing the track irregularity. Overall, compared to the traditional frequency domain analysis method, the order analysis method for identifying track irregularities under non-stationary conditions has significant advantages. Section 6 will further analyze and discuss its application in practical engineering.

## 6. Field Test Case Analysis and Verification

Based on the analysis and derivation in Section 3 and Section 4, using the axle-box vertical vibration acceleration and vehicle running speed to estimate the track vertical irregularity state (wavelength, amplitude) is feasible. This section will conduct analysis and research to apply the above series of methods in practical engineering.

### 6.1. Calculation of Time-Domain Track Irregularity

**Figure 26:**
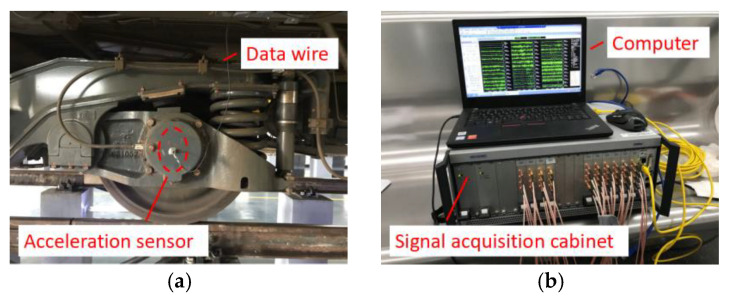
Axle-box vertical vibration acceleration test. (**a**) Acceleration sensor; (**b**) Signal acquisition equipment.

Taking a Chinese subway vehicle as an example, we conducted the axle-box vibration acceleration tests to detect the track irregularity. As shown in Figure 26, glue the acceleration sensor (type: J13510, Shanghai Beizhi Electronic Technology Co., Ltd. (Shanghai, China)) to the axle-box. When the vehicle operates under AW0 (empty load) condition and ATO (automatic train operation) mode, the axle-box vibration acceleration signal is transmitted to the signal acquisition cabinet (type: INV3020D, China Orient Institute of Noise & Vibration, Beijing, China) via the data line. The sampling frequency is 5120 Hz, and the sampling duration is for full process collection.

**Figure 27:**
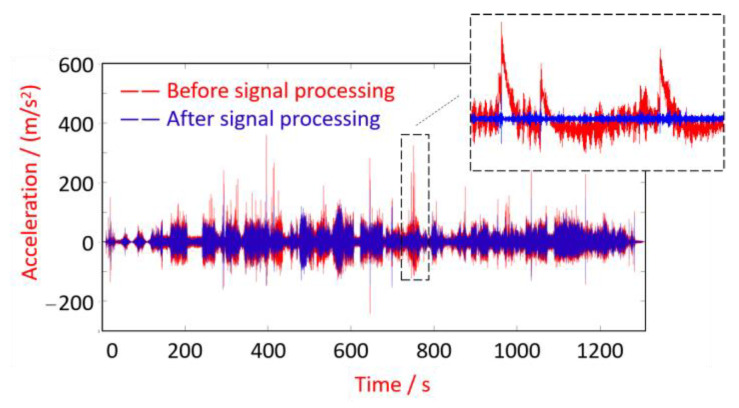
Axle-box vertical vibration acceleration (down-process).

Since the measured signals often have severe test errors and outliers, it is necessary to perform the required pre-processing to the test signals, such as zero drift elimination, singular value elimination, electronic noise elimination, etc. In this paper, based on the signal processing toolbox that comes with MATLAB R2023b, the Savitzky Golay filter smoothing function is called to pre-process the initial signal to eliminate the zero-drift and trend terms due to real-time testing. As shown in Figure 27, the axle-box vibration acceleration signal collected from the down-process shows severe zero-frequency stepwise errors in the local time domain, and the difference before and after signal processing is significant, showing the importance of signal pre-processing.

**Figure 28:**
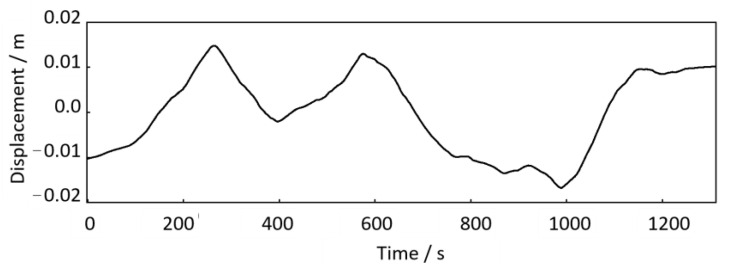
Track irregularity displacement excitation (down-process).

According to the proposed method in Section 3, we performed the time-domain double integration and trend term elimination to calculate the axle-box vibration displacement. Then, according to the transfer relationship *H*(j*ω*) between the axle-box vertical displacement and the track irregularity excitation in Equation (8), the track irregularity displacement excitation *u*(*t*) can be obtained, as shown in Figure 28.

**Figure 29:**
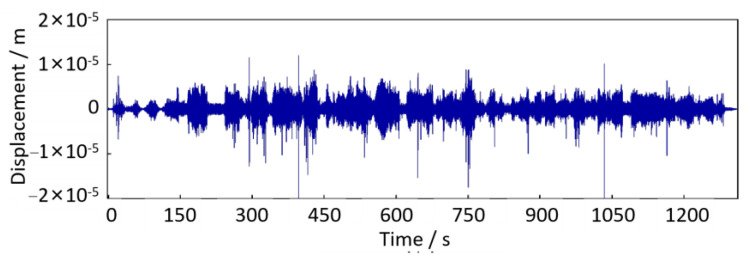
The fluctuation term of track irregularity displacement excitation (down-process).

As shown in Figure 28, there is no apparent short-wave irregularity, which mainly shows height variation throughout the trajectory. This variation can be seen as a low-frequency trend term. The trend and fluctuation terms can be obtained separately by applying the Hodrick–Prescott decomposition method in Section 3. Since the trend term is very close to Figure 28, it is omitted here. The fluctuation term is shown in Figure 29. Compared the axle-box acceleration signal in Figure 27 with the track irregularity displacement excitation in Figure 29, it is found that these two are similar in amplitude and frequency characteristics, with a phase difference of π, proving the correctness of the above calculation method.

### 6.2. Resampling of Time-Domain Displacement Excitation Signal

**Figure 30:**
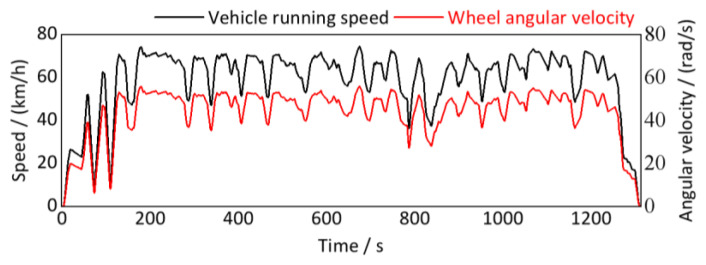
Vehicle running speed and wheel angular velocity (down-process).

Due to the vehicle running at variable speed, the time-domain signal in Figure 29 is non-stationary. However, the traditional signal processing method cannot quantitatively detect the wavelength and amplitude of track irregularities from it. According to the method proposed in Section 4, it is necessary to further resample the time-domain displacement excitation non-uniformly in the equal-angle sample method based on the wheel angular velocity signal and finally achieve quantitative detection of track irregularity through order spectrum analysis. Hence, estimating the wheel angular velocity (rotational speed) signal is critical for equal angle resampling and subsequent order analysis. There are two methods proposed for obtaining wheel angular velocity signals:

**Based on vehicle running speed signal,** rail vehicles usually have their own speed sensors, which can directly derive the real-time vehicle running speed signal from the control system. Then, the wheel angular velocity signals can be converted according to Equation (32), as shown in Figure 30. However, the vehicle running speed derived from the control system is essentially the overall operating speed of the vehicle. Due to the non-rigid connection between vehicles, the overall speed of the vehicle may not represent the real-time angular velocity of a certain wheel, and minor differences may also lead to significant estimation errors. 

**Figure 31:**
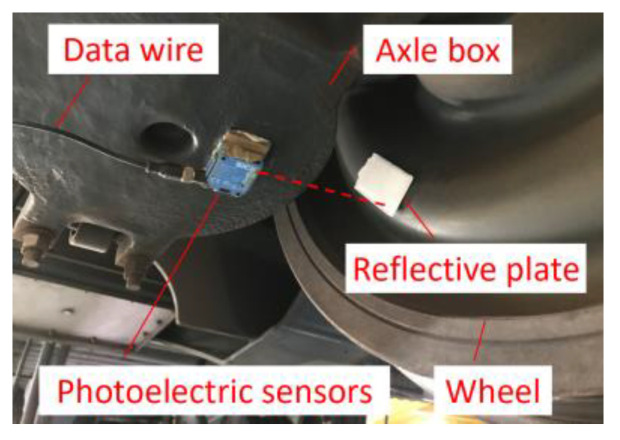
Schematic diagram of the photoelectric sensor installation.

**Based on key-phase signal,** to actually obtain a more realistic real-time wheel rotation angular velocity, the measurement of key-phase signals can be carried out. The key-phase signal is a continuous pulse signal that occurs quantitatively with each wheel revolution and is measured by using the direct beam photoelectric sensor, which is attached to the bottom of the axle-box and aligns the direction of the light source with the wheel as shown in Figure 31. We then attach a reflective plate to the outside of the wheel and align it with the light source emitted by the photoelectric sensor. Finally, we connect the data line of the photoelectric sensor to the signal acquisition box in Figure 26b to collect the continuous photoelectric pulse signal generated during wheel operation.

**Figure 32:**
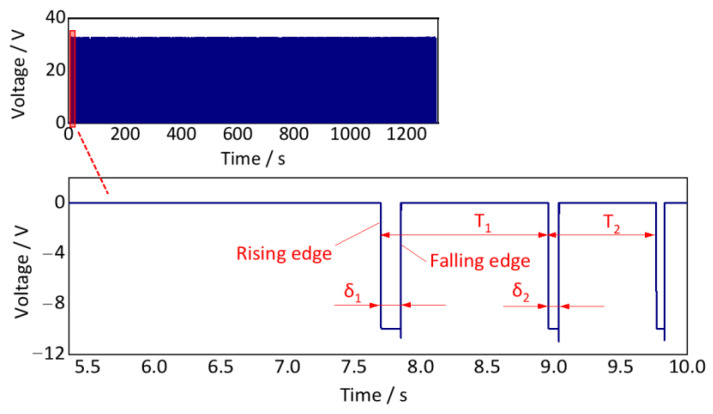
Field test key-phase signal (overall and partial, down-process).

The sampling frequency is essential for the collection of key phase signals. In this case study, the typical operating speed of the subway vehicle is 65 km/h, and the wheel diameter is 0.74 m. The time for the wheels to rotate once is 0.15 s. To ensure the photoelectric sensor can clearly detect the key-phase signals, a higher sampling frequency must be satisfied, as shown in Equation (37). The sampling frequency should be at least 3420 Hz. In this case study, the sampling frequency of the key-phase signal is set to 5120 Hz, which is consistent with the vibration acceleration sampling frequency. It is convenient for later signal processing. The collected key-phase signal is shown in Figure 32.

(37) $$f_{s}=\frac{N}{T_{\min}}$$

where *f_s_* is the sampling frequency, *T*_min_ is the minimum rotation period of the wheel, and *N* is the minimum number of sampling points for one cycle of wheel rotation. When *N* ≥ 500, it can ensure that the signal is almost undistorted.

The key-phase signal is characterized as a rectangular waveform. The interval between two adjacent rising edges (or falling edges) represents one wheel rotation period, as *T*_1_ and *T*_2_, as shown in Figure 32. The pulse widths *δ*_1_ and *δ*_2_ represent the movement time length of the light point emitted by the photoelectric sensor on the reflective white plate. It can be found that the above two adjacent rotation periods *T* and pulse width *δ* are not equal, indicating that the rotation speed of this wheel is non-uniform. Therefore, the axle-box acceleration collected during this period is typically non-stationary. 

Then, using the time point of the rising edge, the wheel rotation speed can be calculated as follows:

(38) $$r_{1\_r}=\frac{60}{k\left(t_{2\_r}-t_{1\_r}\right)}$$

where *t*_1_*r*_ and *t*_2_*r*_ represents the time point of two adjacent rising edges, *T*_1_ = *t*_2_*r*_ − *t*_1_*r*_, *k* is the number of pulses per wheel revolution, *k* = 1; and *r*_1_*r*_ is the wheel rotation speed at time *t*_1_*r*_.

As shown in Equation (38), this calculation method takes the average speed within the time interval [*t*_1_*r*_, *t*_2_*r*_] as the instantaneous velocity at time *t*_1_*r*_. Similarly, the time point of the falling edge also can be used as follows:

(39) $$r_{1\_f}=\frac{60}{k\left(t_{2\_f}-t_{1\_f}\right)}$$

**Figure 33:**
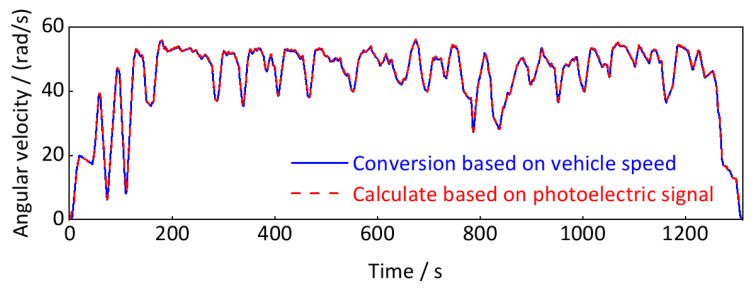
Real-time wheel rotation angular velocity signal (down-process).

Finally, we obtain the instantaneous wheel rotation speed at the time point (*t*_1_*r*_ + *t*_1_*f*_)/2 by calculating the average value of *r*_1_*r*_ and *r*_1_*f*_. According to the conversion relationship between wheel rotation speed and wheel angular velocity in Equation (32), the real-time wheel rotation angular velocity signal calculated, as shown in Figure 33.

As shown in Figure 33, the calculated real-time wheel angular velocity based on the photoelectric key-phase signal is almost identical to that based on vehicle running speed with only some minor fluctuations, indicating the calculation method is theoretically wholly correct.

**Figure 34:**
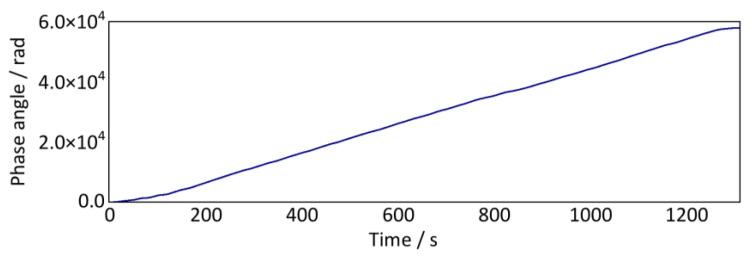
Cumulative phase angle signal of wheel rotation (down-process).

Then, we perform the time-domain integration to the wheel rotation angular velocity signal (Figure 33) to calculate the phase angle time-domain signal, as shown in Figure 34. The accumulated phase angle of the wheel rotation gradually increases with time until the vehicle finally stops. Meanwhile, the phase angle curve does not increase uniformly, which is related to the frequent changes in the vehicle running speed.

According to the relationship between the order *o*, wheel circumference *C*, and track irregularity wavelength *l* (i.e., *o* = *C*/*l*), when the minimum wavelength of concern was 0.05 m, the highest order should be *o*_max_ = *C*/*l*_min_ = 46.5. As shown in Section 4.2, the order spectrum analysis must at least satisfy Δ*O ≤* 0.01, *M* ≥ 2 × 46.5, i.e., *P* ≥ 50, *M* ≥ 93. Meanwhile, a larger M must be considered to avoid the signal aliasing phenomenon and let *M* = 240 ≥ 5 × 46.5 here. Then, *P* is expressed as the number of wheel revolutions. Since the resampling process is necessarily performed on the entire sampled signal, the value of *P* essentially depends on the length of the original sampled signal. The longer the sampling time, the greater the number of wheel revolutions, the greater the value of *P*, which can be expressed as:

(40) $$P=\left\lfloor \frac{\max\left(\theta \right)}{2\pi }\right\rfloor$$

where $\left\lfloor x\right\rfloor$ represents the largest integer less than *x*, and *θ* is the phase angle, as the maximum value in Figure 34.

**Figure 35:**
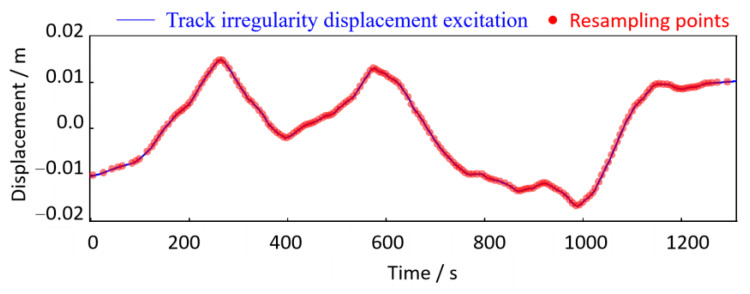
Equal angle resampling points (resampling points are shown at intervals) in the time domain.

**Figure 36:**
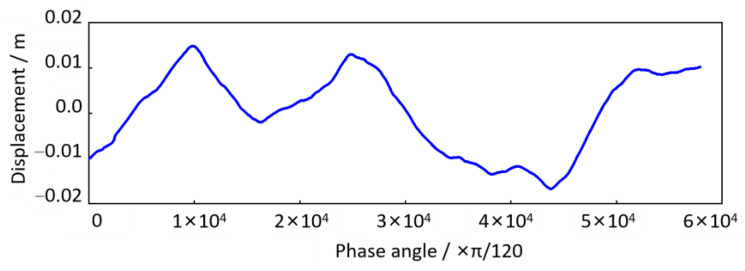
Track irregularity in the angular (spatial) domain.

In this case study, the phase angle of the wheel rotation until the vehicle finally stops is 57,931.05 rad. The *P* ≈ 9220 indicates that the total wheel rotation during the time course in Figure 33 is approximately 9220 revolutions. Hence, the resolution of the order spectrum is Δ*O =* 1/*P =* 0.00010846 *≤* 0.01, which can satisfy the above estimation accuracy requirements. According to the resampling setting conditions above, the time-domain track irregularity displacement excitation in Figure 28 is resampled in the equal-angle sample method (Δ*θ* = 2π/240). As shown in Figure 35, the equal-angle sample result is expressed as non-uniform sampling in the time domain. When the vehicle speed is low, the sampling points are sparse; otherwise, the sampling points are more intensive and can be expressed in the form of an angular domain signal, as shown in Figure 36. The horizontal coordinate is the phase angle of the wheel, and the vertical coordinate is the vertical irregularity amplitude of the track. The fundamental difference between the two types of expression forms is that the track irregularity in the time domain is a non-stationary signal, while in the spatial domain, it is a stationary signal.

### 6.3. Order Spectrum Analysis and Quantitative Detection

**Figure 37:**
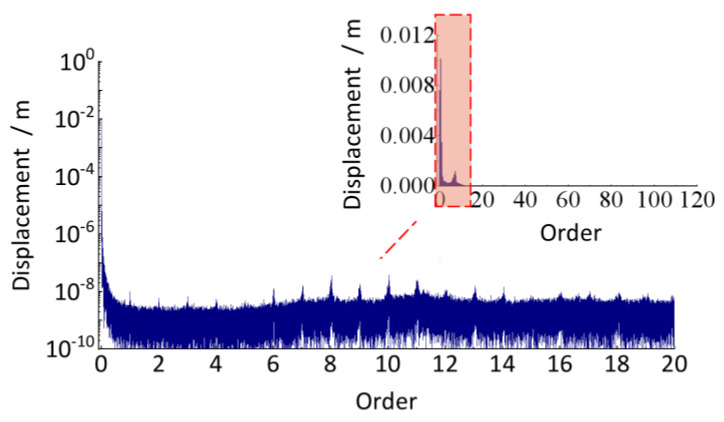
Order spectrum analysis of track irregularity (down-process).

The track vertical irregularity in Figure 36 is a stationary signal in the spatial domain, whose wavelength and amplitude can be detected by discrete Fourier transform. But before that, to avoid the spectrum leakage problem caused by truncation error, refer to the traditional spectrum analysis method, add the Hanning window to it, and then perform spectrum value compensation correction (multiplied by 2.0) for the spectrum result after the discrete Fourier transform. The calculated order analysis spectrum is shown in Figure 37.

Based on the relationship between order, track vertical irregularity wavelength, and wheel circumference, these irregularities with a wavelength less than 116.25 mm correspond to the order over 20th in Figure 37 and have minimal amplitude, indicating the track has a good condition in short-wavelength irregularity wear. The lower order region (less than 1) represents medium and long wave irregularities, whose wavelengths are greater than the wheel’s circumference, with relatively stable overall and no obvious periodic irregularity feature. Hence, this subway track line in service for about 2 years has good quality and is mainly characterized by random irregularity.

It is worth noting that, as shown in Figure 37, the amplitude of irregularities over the 20th order is minimal and has almost no effect on the overall evaluation of track irregularities. It is unnecessary to take *M* = 240 when resampling the time-domain displacement excitation. In the following analysis cases, compared with when *M* = 100, the number of sample points for one wheel revolution is reduced from 240 to 100, significantly improving the resampling efficiency and reducing the computation time from about 32 h to about 16 h.

**Figure 38:**
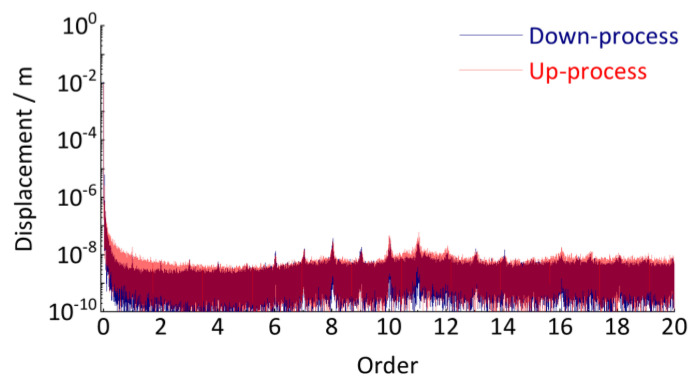
Comparative analysis of detection results for track irregularities.

To verify the accuracy of the quantitative detection method, another set of test data (up-process) was used to calculate the track vertical irregularity of the above subway line again. Due to the difference in operating speed, the axle-box vibration acceleration and key-phase signal generated by the same vehicle running on the same track will also be completely different from the above signals (Figure 27 and Figure 32). If the estimated track irregularity results based on two completely different sets of test data do not differ significantly, it can prove the accuracy and reliability of the proposed method. As shown in Figure 38, the similarity between the estimation results of track irregularity based on the two test results is extremely high, especially the peaks at orders 6, 7, 8, 9, 10, 11, 12, 13, and 14 are almost identical, which fully proves the accuracy and reliability of the proposed method for identifying vertical periodic track irregularity in this paper.

The reasons for the detection error in Figure 38 are as follows. ① During the testing process in this case study, the axle-box vibration signal in Figure 27 and the photoelectric key-phase signal in Figure 32 are collected separately, not absolutely synchronously. These signals are mainly processed manually to align the time starting points of the acceleration and key phase signals, whose errors present in these processes will affect the accuracy of the subsequent resampling. ② The quality of the vibration data collected in this field test is not well. As shown in Figure 27, there was severe step-type zero drift. The introduction of human interference through the moving average processing of the signal will lead to signal distortion. ③ There are some differences in the testing interval. The upward process includes a transition section for exiting the station, which is not included in the downward process. Due to the collection without stopping throughout the process, it is difficult to remove this section accurately. However, overall, the detection results based on the two sets of test data have minimal differences, proving the proposed method’s reliability and accuracy.

## 7. Conclusions

Quantitative detection of track vertical irregularity directly affects the quality and safety of vehicle operation. In this paper, starting from the establishment of a simplified wheel–rail dynamics model, the quantitative relationship between track vertical irregularity excitation and axle-box vertical vibration is theoretically derived in the whole process, and the systematic estimation method and process of track vertical irregularity based on axle-box vertical vibration acceleration is proposed, which mainly involves the following aspects. ① The double time-domain integration of axle-box vertical vibration acceleration by using Simpson’s time-domain integration method is proposed to obtain the axle-box vertical vibration displacement signal, and the calculation error of the method is optimized and improved. ② A method based on the Hodrick–Prescott decomposition is proposed to eliminate the trend term generated in the time-domain integration process. Meanwhile, the optimal value of the smoothing factor *λ* is derived theoretically, and the determination process and the suggested value are proposed; ③ The method and principle of order ratio analysis are derived, analyzed and verified, and finally, the method of estimating the track vertical irregularity based on the axle-box non-stationary vibration signal under the vehicle variable speed operation condition is proposed, which solves the problems of signal non-stationarity, time-domain and space domain conversion, equal angle resampling, and speed estimation based on the photoelectric key signal. 

But it is worth pointing out that: ① the effectiveness of the method proposed in this paper relies on the accurate acquisition of the frequency response function *H*(*ω*) in practice. For the different track types and vehicle system parameters, the frequency response function curves are quite different. ② There is a P2 resonance effect between the vehicle and the track system, indicating it is better to avoid the first wheel–rail resonance peak with low passing frequency to estimate the track vertical irregularity, especially when |*H*(*ω*)| ≈ 1. ③ The lower the operating speed of the vehicle, the wider the wavelength range of track irregularity detection. However, a speed that is too low reduces the detection efficiency. This is a problem that needs to be considered as a compromise. ④ The effect of vehicle lateral motion on the detection results of vertical track irregularities is simplified without considering the weak coupling effect of vehicle lateral motion and vertical motion. It has to be further optimized for practical applications.

## Figures and Tables

**Table 1:** Track irregularity parameters (speed: *v* = 10.8 km/h; wheel circumference: *C* = 2.639 m).

Type	Wavelength *l*/mm	Amplitude *a*/mm	Phase *φ*	Frequency *f*/Hz	Order
Short wave irregularity	50.00	0.30	69.81	60.00	52.78
	200.00	0.60	67.13	15.00	13.19
Medium wave irregularity	1000.00	1.00	17.89	3.00	2.64
	3000.00	2.00	54.55	1.00	0.88
	8000.00	4.00	70.43	0.38	0.33
	15,000.00	5.00	16.52	0.20	0.18
Long wave irregularity	30,000.00	10.00	60.06	0.10	0.09
	45,000.00	12.00	7.25	0.07	0.06
	60,000.00	18.00	88.54	0.05	0.04

**Table 2:** Detection error analysis of track irregularities.

Wavelength *l*/mm		Error (%)	Amplitude *a*/mm		Error (%)
Real Value	Estimated Value		Real Value	Estimated Value	
50.00	50.00	0	0.30	0.30	0
200.00	200.00	0	0.60	0.60	0
1000.00	999.98	0	1.00	1.00	0
3000.00	3002.20	−0.07	2.00	1.70	−17.00
8000.00	7996.78	0.04	4.00	4.00	−0.50
15,000.00	14,909.25	0.61	5.00	4.10	−18.60
30,000.00	30,332.62	−1.1	10.00	8.70	−13.50
45,000.00	45,498.93	−1.1	12.00	10.30	−14.42
60,000.00	58,643.06	2.31	18.00	14.60	−18.72

## Data Availability

Data are contained within the article.
